# Isolation and Characterisation of 1-Alkyl-3-Methylimidazolium Chloride Ionic Liquid-Tolerant and Biodegrading Marine Bacteria

**DOI:** 10.1371/journal.pone.0060806

**Published:** 2013-04-01

**Authors:** Julianne Megaw, Alessandro Busetti, Brendan F. Gilmore

**Affiliations:** School of Pharmacy, Queen’s University of Belfast, Belfast, United Kingdom; Ben-Gurion University of the Negev, Israel

## Abstract

The aim of this study was to isolate and identify marine-derived bacteria which exhibited high tolerance to, and an ability to biodegrade, 1-alkyl-3-methylimidazolium chloride ionic liquids. The salinity and hydrocarbon load of some marine environments may induce selective pressures which enhance the ability of microbes to grow in the presence of these liquid salts. The isolates obtained in this study generally showed a greater ability to grow in the presence of the selected ionic liquids compared to microorganisms described previously, with two marine-derived bacteria, *Rhodococcus erythropolis* and *Brevibacterium sanguinis* growing in concentrations exceeding 1 M 1-ethyl-3-methylimidazolium chloride. The ability of these bacteria to degrade the selected ionic liquids was assessed using High Performance Liquid Chromatography (HPLC), and three were shown to degrade the selected ionic liquids by up to 59% over a 63-day test period. These bacterial isolates represent excellent candidates for further potential applications in the bioremediation of ionic liquid-containing waste or following accidental environmental exposure.

## Introduction

With growing restrictions on acceptable limits of worker exposure and environmental release of carcinogenic, mutagenic, toxic, persistent and bioaccumulative compounds [1], there is an increasing global demand for the adoption of policies of green chemistry and sustainability in research and industry [2], [3]. This has necessitated the search for alternative, safer compounds, provoking the exploration of ionic liquids as viable replacements for many conventional and volatile organic solvents. A major advantage of ionic liquids is their ‘tuneable’ nature, with simple structural modifications enabling many of their physicochemical properties to be altered, which is particularly beneficial in chemical processes which may be limited by the available solvents [4]. Consequently, ionic liquids have found numerous and diverse applications [5]–[8]. Other benefits in terms of operational and environmental safety include their negligible vapour pressure, non-flammability, recyclability and thermostability, which have led to ionic liquids frequently being referred to as ‘green’ solvents.

The growing interest in, and application of ILs in industrial-scale processes may eventually result in environmental exposure, since many are water-soluble and could potentially enter the environment via the contamination of aqueous effluents [9]. It is therefore important to understand not only their potential environmental toxicity, but also their ultimate fate. Given the expanding potential of ionic liquids beyond industrial processes, as typified by their recent exploration as active pharmaceutical ingredients [10], more widespread utilisation of ionic liquids outside laboratories could provide a further potential route of entry into the environment. Despite being characterised as ‘green’ solvents and reaction media, ionic liquids exhibit wide-ranging toxicity, and in some cases have been shown to be more toxic than the solvents for which they are potential replacements [11]–[13]. Toxicity has been observed across a spectrum of organisms, with some potent effects observed even at very low concentrations [13]–[19]. A generally-accepted mechanism of ionic liquid toxicity is via membrane accumulation and disruption and surface activity, with subsequent accumulation within cells [20], [21]. The observed trend of increasing toxicity with ionic liquids containing hydrophobic alkyl chain substituents of increasing chain length holds true for all studies to date, indicating that increased lipophilicity from the longer alkyl substituents facilitates increased membrane interactions, up to an optimal chain length of 12–16 carbon atoms. Though each ionic liquid has its own specific physicochemical and biological properties and consequently will affect organisms in different ways, some of the toxicological effects may not be entirely due to ionic liquid-specific properties, but to general, colligative properties possessed by solutes, for example reduction in water activity (a_w_) of an aqueous solution [22] contributing to osmotic stress, which is known to negatively affect numerous cellular processes [23]. The stability of ionic liquids, which is one of the main advantages in terms of their application in green chemistry, may also prompt concerns for their environmental bioaccumulation. Limited biodegradation of many ionic liquids, described in previous studies [24]–[28] may facilitate their passage through water treatment systems relatively unaltered [25], [29], [30], and coupled with the lipophilicity of many ionic liquids, could also contribute to persistence and accumulation in the environment [20].

The most common currently-available methods of ionic liquid degradation involve advanced oxidation processes such as chemical, photochemical, electrochemical and thermal degradation [31], [32], which are highly effective at degrading contained samples of ionic liquids in a laboratory setting, but may not prove practical or safe options in the event of environmental release. An alternative to chemical degradation methods, and a superior option when addressing the possibility of environmental release, is biodegradation, eliminating the use of harmful chemicals or processes. *In situ* environmental bioremediation has shown potential in the removal of various contaminants from soils and groundwater [33]–[36]. However, ionic liquids are synthetic compounds with presumably negligible environmental exposure to date. The biodegradation of xenobiotic compounds is difficult for many organisms as they may not possess the necessary enzymes to carry out critical steps in a catabolic pathway [37]. However, even xenobiotic compounds can be degraded to an extent if an existing metabolic pathway for similar molecules is present, and as many ionic liquids have some structural analogies with many hydrocarbons, they have proven to be at least partly biodegradable. In keeping with observations relating to toxicity, ionic liquids’ biodegradability is shown to increase with increasing chain length. Because of this, there is an upper limit to the length of chain which can be degraded, as toxicity will eventually overcome biodegradation potential [38], [39]. This is similar to the patterns of alkane toxicity and biodegradability [40], [41], with which some ionic liquids share considerable structural homology. Many studies of ionic liquid toxicity and biodegradability are short-term [28], [30], [42], [43], with fewer published long-term studies [29], [44] hence their long-term toxicological impact and ultimate degradability are not fully addressed.

It is well established that pre-exposure has a profound effect on the ability of microorganisms to degrade certain compounds. Without pre-exposure, extensive acclimation periods may be required before biodegradation occurs, whereas with pre-exposure, the required catabolic activity has already been enriched, resulting in biodegradation occurring readily [45], [46]. It has been observed that hydrocarbon-polluted and saline soils can yield ionic liquid-tolerant and ionic liquid-degrading microorganisms, as these two environments may induce selective survival pressures, enhancing the ability of organisms from that environment to tolerate and degrade ionic liquids [47]. We propose that marine environments are potentially excellent sources for the selective isolation and characterisation of microorganisms exhibiting elevated tolerance to, and an ability to degrade ionic liquids. The marine environment offers unique environmental conditions, with salinity and hydrocarbon load potentially acting as pre-exposure stimuli. Consequently, marine environments have been shown to be a rich source of microorganisms adapted to biodegrade a wide diversity of compounds as nutrient sources [48]–[53].

**Figure 1:**
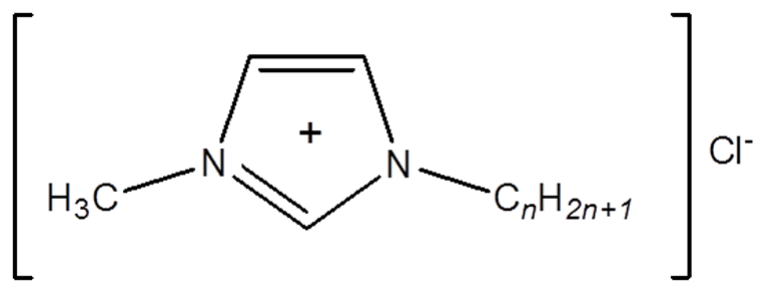
General structure of 1-alkyl-3-methylimidazolium chloride ionic liquids.

We report the isolation and characterisation of marine-derived bacteria exhibiting high tolerance, and importantly, an ability to biodegrade the 1-alkyl-3-methylimidazolium chloride ionic liquids, [C_n_mim]Cl (general structure is given in Figure. 1). Such bacteria may form the basis of our understanding of the environmental impact of ionic liquids and their metabolism, and may prove useful components of a ‘microbiological spill-kit’ in the event of accidental environmental exposure.

## Materials and Methods

### Ionic liquids

1-ethyl, -butyl, -hexyl, -octyl and -decyl -3-methylimidazolium chloride were purchased from Sigma-Aldrich (UK). No solubility issues were observed for any of the ionic liquids used in this study; all compounds were freely soluble in water.

### Isolation, characterisation and identification

Seawater, rockpool water and sand samples were obtained from Kilkeel, Co. Down, Northern Ireland, from a site adjacent to a busy harbour. Isolations were performed on solid M9 minimal salts medium (Sigma-Aldrich, UK) supplemented with [C_n_mim]Cl ranging in concentration from 1–5% w/v as their sole carbon source, LB agar with the same ionic liquid concentrations, and a medium used by Deive *et al*. [47], containing 0.1% peptone and 0.5 or 1 M of the selected ionic liquids. Undiluted water samples were inoculated directly in/onto the media and sand samples were suspended in a small amount of autoclaved seawater which was then directly inoculated. Isolations were carried out at 28 and 37°C, and all bacterial isolates obtained were streaked a minimum of twice on solid media to ensure culture purity. All isolates obtained were characterised by Gram staining and identified by 16S rRNA sequencing using primers 27F (5′-AGAGTTTGATCMTGGCTCAG-3′) and 1492R (5′-TACGGYTACCTTGTTACGACTT-3′).

### MIC/MBC determination

Serial doubling dilutions of each ionic liquid (from an original 0.22 µm sterile filtered working stock) were prepared in 100 µL LB broth in 96-well microtitre plates over the range 0.0000076 – 25% w/v. Each inoculum was prepared by adjusting the turbidity of an actively growing broth culture to an optical density at 550 nm equivalent to 1×10^8^ CFU/ml. This was further diluted to provide a final inoculum density of 2×10^5^ CFU/ml in LB broth which was verified by total viable count. 100 µL of the inoculum was added to each well of the microtitre plates (6 replicates). Positive and negative growth controls were also included in each plate. All plates were incubated at 28°C and 100 rpm for 72 h after which the minimum inhibitory concentration (MIC) (lowest concentration at which no growth was observed) was noted. After MIC determination, the minimum bactericidal concentrations (MBCs) were determined by spreading 20 µL aliquots of media from wells showing no growth onto plates of LB agar, which were then incubated for 48 h at 28°C and examined for 99.9% killing.

### Preliminary screen for [C_n_mim]Cl biodegradation

All isolates (along with another previously-isolated strain) were streaked onto solid M9 minimal salts medium containing [C_n_mim]Cl as their sole carbon source, ranging in concentration from 1–20% w/v. Plates were incubated in sealed containers for up to 8 weeks at 28°C and periodically examined for growth.

### Biodegradation analysis

All isolates were grown overnight in 100 ml LB broth cultures in 250 ml Erlenmeyer flasks at 28 °C. All broth cultures were centrifuged and biodegradation analysis conducted on both cells and supernatants. Supernatants were 0.22 µm sterile filtered and the [C_n_mim]Cl (n = C_2_–C_10_) added from stock solutions in LB broth that were 0.22 µm sterile filtered to achieve a concentration of 1 g L^−1^. Cell pellets were washed to remove residual LBB by resuspending in M9 minimal salts medium containing no carbon source and pelleting again. The M9 medium was discarded after centrifugation and the washed pellets were resuspended in M9 minimal salts medium containing [C_n_mim]Cl (n = C_2_–C_10_). All samples were incubated at 28°C and 100 rpm, and 1 ml samples were removed periodically and analysed by HPLC. Samples containing cells were cleaned by centrifugation at 12000 g for 15 min and then 0.22 µm filtered prior to analysis. The HPLC system used was an Agilent 1260 Infinity equipped with a G1311C quat pump, G1329B autosampler, G1316A column compartment and G1314C variable wavelength detector. The column was a Phenomenex Jupiter 5u C18 300A with dimensions of 250 × 4.6 mm × 5 µm. For analysis of cell suspensions the mobile phase was 75% acetonitrile: 25% 10 mM K_2_HPO_,_ and for supernatants the mobile phase was 80% acetonitrile: 20% 5 mM K2HPO_4_/5 mM H_2_SO_4._ All samples were run isocratically with a flow rate of 1 ml/min. The sample size was 5 µL and all measurements were made at a wavelength of 210 nm, with degradation expressed as a percentage of the control. All analysis was carried out in duplicate and was conducted at least weekly for up to 63 days.

### Biofilm susceptibility assay

Two selected isolates were grown in the Calgary Biofilm Device (commercially available as the MBEC Assay^TM^ for Physiology & Genetics (P & G), Innovotech Inc., Edmonton, Alberta, Canada). The biofilm assay was conducted according to the MBEC^TM^ assay protocol supplied by the manufacturer [54], with slight modifications. Inocula of each organism were prepared in LB broth as described above and adjusted to a final density of 1×10^7^ CFU/ml, as verified by total viable count. 150 µL of each inoculum were transferred to each well of the 96-well microtitre plate packaged with the MBEC assay. The plate lid containing 96 pegs was placed into the microtitre plate and all plates were incubated in a gyrorotary incubator at 28°C and 100 rpm for 24 h. Positive and negative controls were included in each plate (6 replicates). After 24 h, biofilm counts (expressed as CFU/peg) were obtained according to the manufacturer’s instructions. The peg lid of each MBEC plate was rinsed three times a 96-well plate containing 0.9% saline and transferred to a ‘challenge’ plate, each well of which contained 200 µL of LB broth containing the ionic liquids to be tested, prepared by serial doubling dilutions as described above. Positive and negative controls were included in each plate. After 24 hours’ exposure to the challenge plate, the peg lid was removed, rinsed three times in 0.9% saline and transferred into a ‘recovery’ plate with each well containing 200 µL LBB. All plates were sonicated for 15 minutes to dislodge the biofilms into the recovery media and the peg lid was discarded. Recovery plates were incubated for 48 h and visually checked for turbidity, and an MBEC value was assigned as the lowest IL concentration at which no growth was observed after 48 h incubation, which was confirmed by recording optical density measurements at 550 nm.

## Results and Discussion

Fourteen bacterial isolates were obtained using the procedures described above (Table 1), nine on the ionic liquid-supplemented M9 minimal salts medium. As the ILs provided the only available carbon source, any isolates obtained in this manner theoretically had the ability to degrade them. While not used for isolation by Deive *et al.,* their 0.1% peptone medium with 1 M [C_2_mim]Cl [47] yielded one isolate (an immediate indication of high ionic liquid tolerance), which was later identified as *Brevibacterium sanguinis*. The nature of the sampling site - a saline environment in close proximity to a harbour - could account for the presence of ionic liquid-tolerant/biodegrading isolates in this environment. This is in keeping with the findings of Deive *et al.*, who noted that saline and hydrocarbon-polluted soil yielded ionic liquid-tolerant and degrading isolates, when unpolluted soil did not. Taken together, these data indicate that, for biodegradation and tolerance, it is sufficient for ionic liquid naïve microbes to have been previously exposed to compounds structurally analogous to the ionic liquids under test. A strain of *Kocuria palustris* which had been isolated previously from a marine environment was also included in the study as bacteria of the genus *Kocuria* are known for their ability to degrade hydrocarbons [22], [55].

**Table 1:** Bacterial isolates obtained in this study on isolation media containing [C_n_mim]Cl.

Medium	Species and strain, % similarity (NCBI BLAST)
[C_2_mim]Cl M9	*Planococcus donghaensis* IARI-L-39 99% (G+, C)
	*Micrococcus sp*. A-Sh-D-28-1 99% (G+,C)
	*Leucobacter komagatae*115S1 100% (G-, R)
	*Micrococcus yunnanensis* KNUC422 99% (G+, C)
	*Micrococcus luteus* BGCC 1079 100% (G+, C)
[C_4_mim]Cl M9	*Micrococcus luteus* CMS197 99% (G+, C)
	*Micrococcus luteus* czh-8C 99% (G+, C)
	*Rhodococcus erythropolis* XP 99% (G+, R)
	*Rhodococcus fascians* B-G-PYD5 99% (G+, R)
	*Bacillus amyloliquefaciens* FZB42 99% (G-, R)
[C_2_mim]Cl LB	*Exiguobacterium oxidotolerans* CJ-G-PYD7; 99% (G-, R)
[C_4_mim]Cl LB	Arctic seawater bacterium Bsw20350 99% (G-, R)
	*Planococcus donghaensis* MPA1U2 99% (G+, C)
1 M [C_2_mim]Cl + 0.1% peptone	*Brevibacterium sanguinis* CJ-S-TSA3 99% (G+, R)

G+, Gram positive; G-, Gram negative; C, cocci; R, rods.

Many of the isolates proved to be considerably more tolerant to [C_n_mim]Cl than bacteria which have previously described [21], with the majority having higher MIC values than previously reported. We have identified two isolates, *Rhodococcus erythropolis* and *B. sanguinis,* which exhibit exceptionally high tolerance to [C_n_mim]Cl. The fact that *B. sanguinis* was isolated in a medium containing 1 M [C_2_mim]Cl indicates a remarkable inherent tolerance to this ionic liquid, potentially acquired as a result of hydrocarbon pre-exposure in the marine environment. The results obtained for MIC and MBC screening (Table 2) were consistent with numerous studies showing that IL toxicity increases with increasing alkyl chain length [11], [25], [56], due to the linear relationship between chain length and hydrophobicity [57], resulting in a greater ability of longer-chain ILs to intercalate into the cell membrane. As expected, and as reported previously, low toxicity was observed with ILs with shorter (C_2_–C_4_) alkyl chains [15], [58], with antimicrobial effects only observed at very high concentrations. If concentrations are sufficiently high, it is suggested that other factors may contribute to any antimicrobial activity observed, such as solute stress imposed by the excessive concentration of the ionic liquid required for an MIC value to be obtained, in which case even relatively benign ionic liquids may have an antimicrobial effect [47]. The MIC values for *R. erythropolis* and *B. sanguinis* in [C_2_mim]Cl were 1.364 M (20% w/v), with observable growth at the next lowest concentration of 16% w/v (1.09 M) after 48 h. However, both isolates recovered from exposure to 20% w/v [C_2_mim]Cl after inoculation onto recovery media containing no ionic liquid (Table 2). To date, this is the highest concentration of [C_2_mim]Cl at which active growth of a prokaryote has been reported. Deive *et al*. [47] observed no active bacterial growth at concentrations of this ionic liquid exceeding 1 M, but did observe some fungal growth. The ability of these two isolates to actively grow in a medium containing over 1 M [C_2_mim]Cl is most likely in part due to their adaptation to a saline environment. Marine microbes are more likely to be halophilic or halotolerant and therefore well-equipped to deal with this particular environmental stress. *Rhodococcus* and *Brevibacterium spp.* have been shown previously to be tolerant to saline conditions [59], [60].

**Table 2:** MIC and MBC values (mM) of [C_n_mim]Cl against marine-derived bacterial isolates.

	*n*					
Isolate		2	4	6	8	10
*P. donghaensis*	**MIC**	273	57	25	3	0.30
IARI-L-39	**MBC**	273	115	25	3	0.30
*Micrococcus sp*.	**MIC**	546	229	99	5	0.60
A-Sh-D-28-1	**MBC**	1091	458	99	22	2.41
*L. komagatae*	**MIC**	1091	458	197	11	1.21
115S1	**MBC**	1705	916	197	11	2.41
*M. yunnanensis*	**MIC**	546	229	99	11	0.60
KNUC422	**MBC**	1705	916	197	22	1.21
*M. luteus*	**MIC**	1091	458	197	11	1.21
BGCC 1079	**MBC**	1705	916	197	22	2.41
*M. luteus*	**MIC**	546	229	99	11	0.60
CMS197	**MBC**	546	229	99	11	2.41
*M. luteus*	**MIC**	1091	229	197	5	0.60
czh-8C	**MBC**	1091	229	197	5	1.09
*R. erythropolis*	**MIC**	1364	916	197	11	1.21
XP	**MBC**	1705	916	197	11	2.41
*R. fascians*	**MIC**	1091	229	49	3	0.60
B-G-PYD5	**MBC**	1705	458	99	3	0.60
*B. amyloliquefaciens*	**MIC**	1091	229	25	3	0.30
FZB42	**MBC**	1705	458	99	5	9.66
*E. oxidotolerans*	**MIC**	546	229	99	3	0.30
CJ-G-PYD7	**MBC**	1364	229	99	5	2.41
Arctic seawater bacterium	**MIC**	1091	229	99	5	0.60
Bsw20350	**MBC**	1091	229	99	5	0.60
*P. donghaensis*	**MIC**	68	29	12	1	0.08
MPA1U2	**MBC**	136	29	12	3	0.08
*B. sanguinis*	**MIC**	1364	458	197	11	1.21
CJ-S-TSA3	**MBC**	1705	916	197	11	2.41
*K. palustris*	**MIC**	1091	916	49	1	0.15
M16_2A	**MBC**	1091	916	49	1	0.15

**Figure 2:**
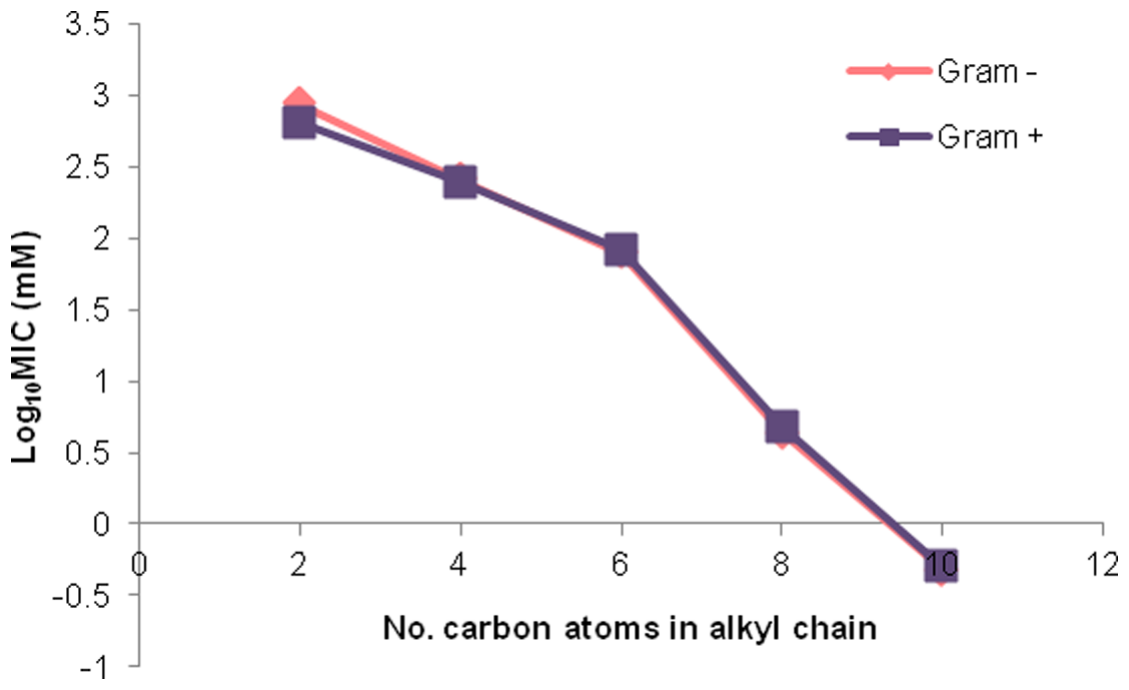
Mean [C*_n_*mim]Cl minimum inhibitory concentration (MIC) values for Gram positive and Gram-negative marine-derived bacterial isolates.

The MIC values were calculated as log_10_ mM and are plotted in Fig. 2 to indicate the relationship between the length of the alkyl chain and log_10_ MIC (mM). Interestingly there was no discernible difference between the MIC values obtained for Gram-positive and Gram negative isolates. This was unexpected as previous reports on the microbiological toxicity of [C_n_mim]Cls [21], [58] indicated that Gram positive bacteria are generally more sensitive to imidazolium ionic liquids than corresponding Gram negative species. The lack of difference in tolerance profile observed between Gram positives and Gram negatives in this study may possibly reflect the sampling and isolation methods used, which deliberately select for highly-tolerant bacteria, irrespective of morphology.

Three of the isolates (*B. sanguinis*, *R. erythropolis* and *K. palustris*) grew particularly well on the ionic liquid-supplemented M9 minimal salts medium, showing dense growth by day 28 (Table 3), and elevated tolerance as shown in Table 2. This suggests that these isolates had the capacity to directly metabolise the ionic liquids present as a sole carbon source quite efficiently. Whilst little is currently known about *Brevibacterium sanguinis* (with only one report in the literature, describing its isolation from a blood sample) [61], there are numerous reports of closely-related bacteria of the genus *Brevibacterium* degrading a wide array of hydrocarbon-containing compounds as a sole carbon source such as biphenyl, phenanthrene, polycyclic aromatic hydrocarbons (PAHs) such as acenapthene, anthracene, fluorene, fluranthene, naphthalene and pyrene, branched and straight-chain alkanes, and crude oil [62]–[65]. Bacteria in the genus *Rhodococcus* are known for their ability to biodegrade numerous compounds [66], with *R. erythropolis* having been shown to degrade a remarkable range of different compounds as a sole carbon source using a wide variety of mechanisms [67], including degradation of n-alkanes when in a minimal salts medium [68]. *K. palustris* has been shown to be able to degrade petroleum hydrocarbons [69]. Tolerance to the selected compounds is of primary importance before biodegradative ability is addressed, as to degrade a compound in useful quantities, the organism must first be able to tolerate and maintain cellular function in its presence. The copious growth of these three isolates on the minimal media, their remarkable tolerance (as indicated by high MIC values) for these compounds, in addition to strong evidence in the literature indicating their proficient hydrocarbon-biodegrading ability, correlate well with their ability to biodegrade these compounds *in vitro*. The majority of the isolates in this study, however, grew poorly or not at all on the M9 media despite many having been isolated on them. It is likely that these isolates exhibited initial tolerance to these compounds, but could not biodegrade or metabolise them efficiently enough to maintain their growth long term. A residual amount of carbon present in the medium (for example residual nutrients carried over from the original sample) may have facilitated the initial meagre growth observed.

**Table 3:** Growth characteristics of marine-derived bacterial isolates on plates containing M9 minimal salts medium containing [C*_n_*mim]Cl as the sole carbon source.

n	2			4		6		8	
(% w/v)	5	10	15	20	5	10	1	5	1
P. donghaensis IARI-L-39	–	–	–	–	–	–	–	–	–
Micrococcus sp. A-Sh-D-28-1	+	+	–	–	+	–	+	–	–
L. komagatae 115S1	+	+	–	–	+	–	+	–	–
M. yunnanensis KNUC422	+	+	–	–	+	–	+	–	–
M. luteus BGCC 1079	+	+	–	–	+	–	+	–	–
M. luteus CMS197	+	–	–	–	+	–	+	–	–
M. luteus czh-8C	+	+	–	–	+	–	+	–	–
R. erythropolis XP	+++	++	+	+	++	–	++	–	–
R. fascians B-G-PYD5	+	+	–	–	+	–	+	–	–
B. amyloliquefaciens FZB42	–	–	–	–	–	–	–	–	–
E. oxidotolerans CJ-G-PYD7	–	–	–	–	–	–	–	–	–
Arctic seawater bacterium Bsw20350	+	–	–	–	–	–	–	–	–
P. donghaensis MPA1U2	–	–	–	–	–	–	–	–	–
B. sanguinis CJ-S-TSA3	+++	++	+	+	++	–	++	–	–
K. palustris M16_2A	++	+	+	–	+	–	+	–	–

Slight growth (+), moderate growth (++), dense growth (+++).

**Figure 3:**
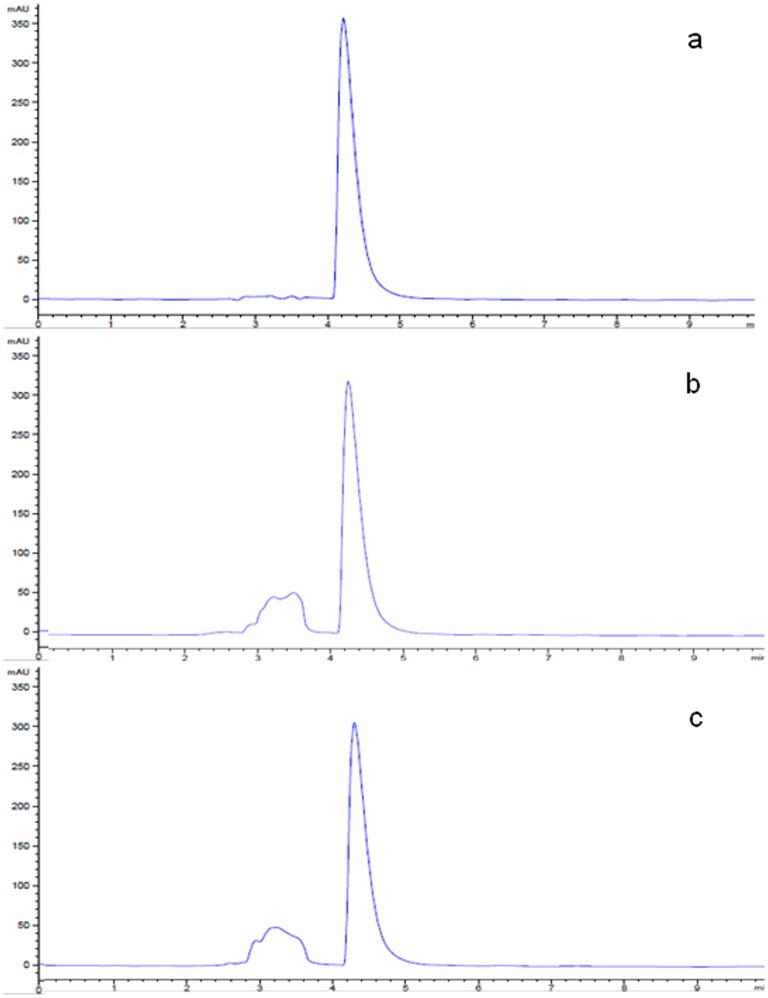
Biodegradation analysis of [C_4_mim]Cl in M9 minimal salts medium by selected isolates after 7 days. Chromatograms presented show (a) uninoculated control medium, (b) *B. sanguinis*, and (c) *R. erythropolis*.

**Figure 4:**
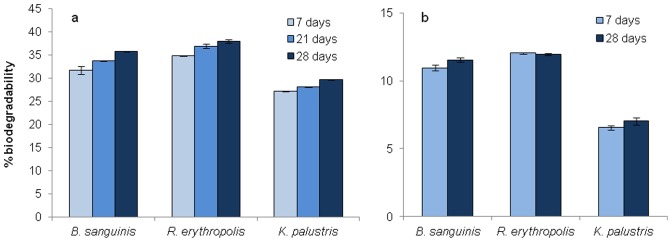
Biodegradation of (a) [C_2_mim]Cl, and (b) [C_4_mim]Cl by cell suspensions of *B. sanguinis, R. erythropolis*, and *K. palustris* after 7, 21, and 28 days. Plotted values are the mean of duplicate measurements; error bars represent one standard deviation.

Planktonic suspensions of three isolates (*B. sanguinis, R. erythropolis* and *K. palustris*) exhibited a moderate ability to degrade [C_n_mim]Cl ionic liquids with alkyl chains of C_2_ and C_4_, observed by HPLC analysis of the growth media as a reduction in the major ionic liquid peak and the emergence of a secondary peak (Figure 3). Part of the reduction in the major peak could be explained by cellular sorption and uptake/sequestering into the cells [20], however, the emergence of a secondary peak in the HPLC trace indicates degradation of the parent compound and evolution of a metabolic by-product. Attempts were made to deduce the composition of the secondary peaks but unfortunately these proved unsuccessful. The data obtained from the planktonic suspension degradation study corresponded with the results obtained in the preliminary screen on solid media as shown in Table 3, with those bacteria showing greatest ability to grow on the M9 plates showing the greatest capacity to degrade the ionic liquids. After 28 days, *B. sanguinis, R. erythropolis* and *K. palustris* degraded [C_2_mim]Cl to 36.5, 38.8 and 29.6% of the control respectively, and [C_4_mim]Cl to 11.5, 12.0, and 7.0% respectively (Figure 4), no degradation of ionic liquids bearing larger alkyl substituents was observed. This is contrary to the increase in degradation with the increase in chain length (until the limit of toxicity is reached) that was expected [24], [26], [70] but is in keeping with the results obtained by Abrusci *et al.,* who found that increasing chain length reduced biodegradability [42]. The other isolates showed no or negligible levels of biodegradation of any of the tested ILs and even with the three positive isolates, there was no degradation at alkyl chain lengths above C_4_. A possible reason for the lack of biodegradation above C_4_ may be due to suboptimal physiological conditions following prolonged incubation in a nutrient-poor medium with a poorly-accessible carbon source, resulting in these isolates exhibiting greater susceptibility to the toxic effects of the ionic liquids of increased alkyl chain length, than might be predicted.

**Figure 5:**
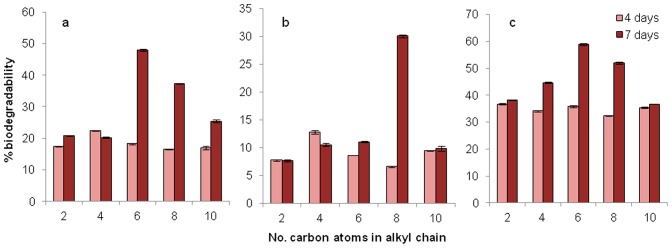
Biodegradation of [C_n_mim]Cl by supernatants of (a) *B. sanguinis,* (b) *R. erythropolis* and (c) *K. palustris*, after 4 and 7 days. Plotted values are the mean of duplicate measurements; error bars represent one standard deviation.

After 7 days, in the supernatants with added [C_n_mim]Cl, greater biodegradation, compared to cell suspensions, was generally observed, with all ionic liquids tested (up to C_10_) being partially degraded (Figure 5). Supernatants of *K. palustris* exhibited greatest biodegradative capacity, degrading 1-ethyl, -butyl, -hexyl, -octyl and -decyl -3-methylimidazolium chloride by 38.2, 44.7, 58.9, 51.9 and 36.7% respectively. In this case, the expected increase in biodegradability was observed with increasing chain length, with the maximum degradation observed at a chain length of C_6_ for *B. sanguinis* and *K. palustris*, and C_8_ for *R. erythropolis*, after which biodegradation decreased. This follows the pattern expected for hydrocarbon degradation, consistent with the results of Whyte *et al*., who observed that alkane degradation was optimal with alkyl chain lengths of C_5_–C_8_ compared to a severely-reduced biodegradation of alkyl chain lengths of C_10_ and above [40], with minimal biodegradation of longer alkyl chains due to predominating toxic effects. These data suggest that the alkyl chain substituent is being degraded, and hence could account for the incomplete biodegradation observed.

Supernatant-mediated biodegradation occurred over a larger range of ionic liquids than with the cell suspensions, most likely because all secreted molecules and enzymes remained in the medium. As the cell pellets were thoroughly washed before addition of the M9 minimal medium, any secreted enzymes, plus nutrients and carbon sources were removed, and only isolates with the ability to regrow in the minimal medium would be able to produce them again, most likely reducing their overall ability to degrade the ionic liquids in comparison to the supernatants. It has been shown that bioremediation efficiency can be enhanced when additional carbon sources are present, especially if the xenobiotic compound alone cannot provide sufficient energy, in addition to enhancing tolerance to the target compounds [71], [72]. This may account for the increased biodegradation observed in the supernatants. Furthermore, this also suggests that biodegradation in the minimal media could potentially be improved if an additional carbon source was added. In all cases, the majority of the degradation occurred within the first 7 days. Although the study was continued for 63 days, minimal further degradation was observed. The biodegradation efficiencies reported in this study are comparable and in some cases superior to those previously described in the literature (summarised in Table 4).

**Table 4:** A summary of previously-reported microbial biodegradation efficiencies of methylimidazolium-based ionic liquids.

Reference	Compound	Biodegradation (%)	Duration	Organism
[29]	[C_2_mim]Cl	0	328 d	Activated sludge
	[C_8_mim]Cl	0		
[30]	[C_2_mim]Cl	0	31 d	Activated sludge
	[C_4_mim]Cl	0		
	[C_6_mim]Cl	8		
	[C_8_mim]Cl	100		
[42]	[C_2_mim]Cl	53	28 d	*Sphingomonas*
	[C_4_mim]Cl	39		*paucimobilis*
	[C_6_mim]Cl	37		
	[C_8_mim]Cl	32		
[47]	[C_2_mim]Cl	0	2 months	Salt marsh soil
	[C_4_mim]Cl	0		isolates

The three isolates which exhibited the greatest ability to biodegrade the selected ionic liquids were examined for their ability to form biofilms and the biofilms’ tolerance to the same ionic liquid challenges as the planktonic cells were examined. A common feature of biofilms is their greatly enhanced tolerance to antimicrobial challenges compared to planktonic bacteria of the same species [73]–[75]. Preliminary experiments using the MBEC device showed that *K*. *palustris* was unable to consistently form biofilms and was excluded from the biofilm susceptibility assay. The MBEC values obtained for the other two isolates were much lower than expected, with both MBEC values equivalent to, or one doubling dilution higher than the MIC (data not shown). It has been previously shown that for [C_n_mim]Cl the MBEC values for a range of other bacteria were significantly higher than the MIC [58] which suggests that for these two isolates, at concentrations exceeding the MIC, the integrity of the biofilm cannot be maintained or provides no additional tolerance benefit. Immobilising these bacteria as a permanent biofilm, for example in a membrane reactor, would provide a suitable interface for ionic liquid biodegradation. It has been shown that biofilm bioreactors can efficiently degrade many compounds more effectively than their planktonic counterparts [76]. As the MIC and MBC values of the *R. erythropolis* and *B. sanguinis* isolates were exceptionally high, it is unlikely that environmental concentrations would reach this value, even in the event of an accidental exposure/spill. Therefore, as long as the IL concentration does not exceed the MIC, biodegrading bacteria such as those described here could effectively be grown as biofilms in any potential ionic liquid bioremediation reactor.

## Conclusions

The isolation of microorganisms from the environment using minimal media, where the sole carbon sources are 1-alkyl-3-methylimidazolium chloride ionic liquids, is an effective and facile method for the selection of bacteria which exhibit an ability to biodegrade or tolerate high concentrations of these compounds, as evidenced by their rapid growth in concentrations exceeding those previously described in the literature. This is likely due to selective pressures imposed by the conditions in the environment from which they were isolated. The three isolates which were identified as being particularly effective ionic liquid biodegraders are potential candidates for the remediation of 1-ethyl- and 1-butyl-3-methylimidazolium chlorides. The ability of two of these bacteria to form biofilms may also provide good candidate organisms for further studies of the role of biofilms in the ultimate bioremediation of ionic liquid-containing waste.
